# Satisfaction and Preferences for Infusion Therapies in Advanced Parkinson’s Disease—Patient Perspective

**DOI:** 10.3390/medicina61010027

**Published:** 2024-12-28

**Authors:** Julia Węgrzynek-Gallina, Tomasz Chmiela, Michał Borończyk, Aleksandra Buczek, Patrycja Hudzińska, Hubert Bigajski, Damian Waksmundzki, Justyna Gawryluk, Joanna Siuda

**Affiliations:** 1 Department of Neurology, Faculty of Medical Sciences in Katowice, University Clinical Centre Prof K. Gibinski, Medical University of Silesia, 14 Medykow St. 40-752 Katowice, Poland; d201258@365.sum.edu.pl (J.W.-G.); tchmiela@sum.edu.pl (T.C.); jgawryluk@sum.edu.pl (J.G.); 2 Department of Neurology, School of Health Sciences, Medical University of Silesia in Katowice, 45/47 Ziolowa St., 40-635 Katowice, Poland; mboronczyk@onet.pl; 3 Students’ Scientific Association, Department of Neurology, Faculty of Medical Sciences in Katowice, Medical University of Silesia, 14 Medykow St, 40-752 Katowice, Poland; s80780@365.sum.edu.pl (A.B.); s80940@365.sum.edu.pl (P.H.); s81699@365.sum.edu.pl (H.B.); s76505@365.sum.edu.pl (D.W.)

**Keywords:** Parkinson’s disease, infusion therapies, levodopa–carbidopa intestinal gel, continuous subcutaneous apomorphine infusion, patients’ preferences

## Abstract

*Background and Objectives:* The rapid growth of the number of advanced Parkinson’s disease (PD) patients has caused a significant increase in the use of device-aided therapies (DATs), including levodopa–carbidopa intestinal gel (LCIG) and continuous subcutaneous apomorphine infusion (CSAI). The objective of this study was to evaluate patients’ satisfaction and the factors influencing preferences for CSAI and LCIG. *Materials and Methods:* The research focused on individuals diagnosed with advanced PD undergoing DAT at the Neurology Department of the University Hospital in Katowice. A telephone survey conducted between June and July 2024 evaluated the experiences of patients with LCIG and CSAI. The Parkinson’s Disease Questionnaire (PDQ-8) and the Stress Scale for Family Caregivers (BSFC-s) were applied. Based on medical record data comprising reasons for the exclusion of individuals, disease-related and treatment data were collected. *Results:* Among the original cohort of 64 patients, 50 completed the survey, including 31 who might choose between infusion therapies. The average patient ages were 70.6 ± 4.7 (CSAI) and 71.2 ± 7.2 years (LCIG), with disease durations of 15 (IQR: 12–19) and 18 (IQR: 13–19) years, respectively. LCIG patients presented higher PDQ-8 scores (20 (IQR: 13–27) vs. 13 (IQR: 6–19), *p* = 0.008), and higher BSFC-s scores (19 (IQR: 12–21) vs. 9 (IQR: 2.5–13), *p* = 0.011). Furthermore, significant factors influencing patient preferences included fear of surgery (75% vs. 36.8%, *p* = 0.043) and concerns about DAT safety (83.3% vs. 47.4%, *p* = 0.049). *Conclusions:* LCIG and CSAI therapies offer benefits and disadvantages, with safety concerns and fear of surgery seeming to be decisive in the decision-making process.

## 1. Introduction

Parkinson’s disease (PD) is a progressive neurodegenerative condition characterized by significant impairment in patients’ daily activities and the potential for various motor complications, including bradykinesia, tremors, muscle rigidity, postural instability leading to falls, and non-motor complications, such as cognitive deficits, autonomic dysfunction, sensory impairments, and sleep disturbances [1,2,3]. PD represents one of the fastest-growing neurological disorders, evidenced by its contribution of 3.2 million disability-adjusted life years (DALYs) in 2016, and a 2.4-fold increase in the number of affected individuals since 1990 [4,5]. Especially, in the advanced stages of the disease, pharmacological treatment becomes insufficient and demands changing the approach [6,7]. Consequently, the utilization of device-aided therapies (DATs), dedicated to advanced PD, is on a consistent upward trajectory.

The DATs currently available in Poland encompass deep brain stimulation (DBS), levodopa–carbidopa intestinal gel (LCIG), continuous subcutaneous apomorphine infusion (CSAI), and foslevodopa/foscarbidopa subcutaneous infusion (LDP/CDP). DBS therapy involves implanting electrodes into the subthalamic nuclei (STN) or the globus pallidus internus (GPi) regions, and implantable pulse generator (IPG) during surgery to modulate electrical impulses in those specific brain targets to improve motor functions in PD patients. LCIG is a continuous infusion of levodopa–carbidopa gel via a portable pump through a percutaneous gastro-jejunostomy tube (PEG-J), usually given 16 h a day with a night break. CSAI and LDP/CDP are subcutaneous treatment methods, with CSAI using apomorphine and the newest method, LDP/CDP, involving a 24 h foslevodopa/foscarbidopa subcutaneous infusion [6].

Presently, none of these therapies reign as superior to the others, with each presenting distinct advantages and disadvantages [6,7]. Consequently, patients may select the most suitable therapeutic approach, a decision that necessitates considering their personal preferences in collaboration with their attending neurologist. It is worth highlighting that most patients express a preference for active involvement in the decision-making process [8,9]. Furthermore, the selection of a specific method is frequently influenced by the patient’s clinical profile, with one approach potentially proving more beneficial than others [10].

When evaluating advanced treatment approaches, it is crucial to consider potential contraindications for a given method. The advantages of infusion therapies (LCIG and CSAI) are manifold and include the eligibility of most patients, the possibility of treatment testing, and, in the case of CSAI, the absence of the need for surgery and its ease of reversibility.

However, infusion therapies also present disadvantages, such as the requirement for continuous wear and operation of the pump, as well as the necessity to attend to the areas on the skin where the cannula is inserted, including the changing of the needle in the case of CSAI [11,12,13], which could pose significant challenges for certain patients. Our study aimed to evaluate satisfaction with infusion therapies and the factors influencing preferences for selecting between CSAI and LCIG in patients with PD (LDP/CDP was not available in Poland when this study was conducted), with particular emphasis on the patient perspective. We hypothesize that patients’ preferences vary depending on the type of infusion therapy.

## 2. Materials and Methods

A single-center survey study was administered to a group of advanced PD patients who use infusion therapies in the Neurology Department in the University Clinical Hospital in Katowice. The list of patients was prepared based on medical records. As the next step, a telephone survey was carried out. Study participation was voluntary and anonymous. The survey was approved by the bioethics committee (BNW/NWN/0052/KB/112/24).

### 2.1. The Patient Selection Process

**Figure 1:**
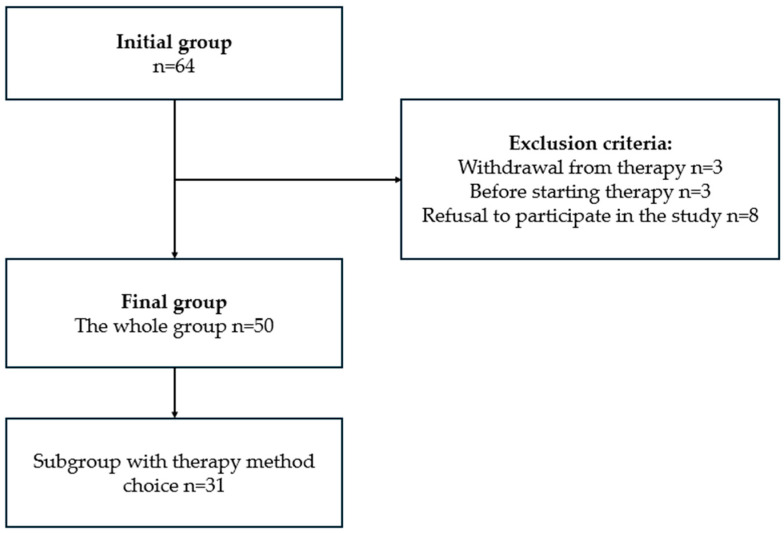
Process of selecting PD patients undergoing infusion therapies.

In the beginning, the researchers prepared a list of 64 patients with advanced PD qualified for infusion therapies based on medical records. There were six people excluded due to either resignation from the infusion therapy before the survey was performed (3) or not yet receiving the infusion therapy but registered in medical records. Based on information from medical records, among 58 patients, there were 31 who were eligible and qualified for both LCIG and CSAI, and might choose therapy according to their preferences. During the telephone survey, an additional eight patients (all qualified only for one therapy) were excluded due to lack of consent to participation. A group of 50 patients was included in the final analysis. The patient selection process is presented in detail in Figure 1.

### 2.2. Patient Survey Assessment

A structured telephone questionnaire was conducted from June to July 2024. It concerned the experience and satisfaction with the treatment of LCIG and CSAI. The questions in the survey focused on the patient demographic data, information about their current infusion therapy and its usage, as well as the therapy’s effectiveness and the patient’s feedback. The survey is presented in Supplementary Materials. For patients who could be qualified for more than one method, an assessment of their preferences was additionally performed. To evaluate the quality of life, Parkinson’s Disease Questionnaire-8 (PDQ-8) was administered via telephone survey. It is a self-administered questionnaire used to measure the quality of life in people with Parkinson’s disease [14]. Moreover, an assessment of caregiver burden by the Short Version of the Buren Scale for Family Caregivers (BSFC-s) was performed. BSFC-s is a screening tool, consisting of 10 questions, created for measuring subjective burden in informal caregivers [15]. Due to greater reliability the currently used therapy, reasons for exclusion from advanced therapies, disease-related data such as disease duration and motor assessment in Movement Disorders Society—Unified Parkinson’s Disease Rating Scale (MDS-UPDRS) part III at the moment of beginning the therapy measured in the ON and OFF states, and date of applying the infusion therapy were supplemented from medical records.

### 2.3. The Statistical Analysis

The statistical analysis was performed with Statistica 13.3 (TIBCO Software Inc. (2017) Statistica (data analysis software system, version 13. https://docs.tibco.com/products/spotfire-statistica/archive, accessed on 27 December 2024)). The quantitative variables are presented as an arithmetic mean and a standard deviation or median and interquartile ranges (IQR). Qualitative variables are presented as absolute values and percentages. The normality of distribution was assessed with the Shapiro–Wilk test. For normal distribution, the intergroup differences for the quantitative variable were evaluated with Student’s t-test. Since the normal distribution in the analyzed groups was not confirmed, the intergroup differences for the quantitative variable were assessed with the U-Mann–Whitney or the Kruskal–Wallis test (variables of skewed distribution). In the case of statistically significant differences within many groups revealed by the Kruskal–Wallis test, a post hoc type of analysis was performed. Fisher’s exact test or Chi-square test was performed for qualitative variables.

## 3. Results

### 3.1. General Study Group

The study group comprised 50 patients: 31 (62%) patients with the possibility to choose between two infusion methods of therapy and 19 (38%) eligible for only one of the two available at that time methods. Among them, 15 (30%) of the patients were treated by CSAI and 35 (70%) used LCIG. Among the surveyed study group, there were 33 (66%) patients who had contraindications to DBS due to cognitive impairment or dementia (24, 72.7%), significant structural brain damage (e.g. after stroke) (8, 24.2%), inappropriate age (over 70 years old) (4, 12.1%), depressive disorders (2, 6.1%) or severe speech disorder (2, 6.1%). The remaining 17 patients were not interested in DBS therapy due to a fear of neurosurgery (9, 52.9%), or preferred infusion therapies (8, 47.1%).

According to contraindications to infusion therapy, 16 (94.1%) people reported that they could not use CSAI due to side effects after using oral dopamine receptor agonists (DA) and 1 (5.9%) reported an insufficient effect of DA therapy. Only 2 people presented contraindications to LCIG due to gastrointestinal disease (1, 50%) and poor drug tolerance (1, 50%).

The mean age of the whole study group was 70.9 ± 6.1 (ranged 61–84) years. Twenty-eight (66%) were males and twenty-two (44%) females. The median duration of PD was 15.5 (IQR: 12–19) years. The median time of DAT was 2 (IQR: 1–4) years. The mean score of the Movement Disorder Society-Unified Parkinson’s Disease Rating Scale (MDS-UPDRS) part III before applying infusion therapy was 55.7 ± 14.8 (OFF) and 29.7 ± 10.4 (ON) for CSAI, and 56.9 ± 15.0 (OFF) and 29.7 ± 9.8 (ON) for the LCIG group. There were no statistical differences between the groups.

The results of the telephone survey showed that the PDQ-8 for PD patients with CSAI was significantly lower than for LCIG (13 (IQR: 6–19) vs. 20 (IQR: 13–27), *p* = 0.008). According to BSFC-s, caregivers assessed LCIG as more burdening than CSAI (9 (IQR: 2.5–13) vs. 19 (IQR: 12–21), *p* = 0.011). Other differences between the groups were not statistically significant. Detailed characteristic of the study group divided into groups was presented in Table 1.

According to the opinion of the patients using infusion therapies, sufficient control of the symptoms was declared by 60% of CSAI users and 74.3% of patients treated with LCIG. Significantly more patients treated with LCIG reported 6–10 h per day reduction in symptoms of PD (13 (37.1%) vs.1 (6.7%), *p* = 0.023), and more patients with CSAI reported less than 4 h (4 (26.7%) vs. 1 (2.9%), *p* = 0.024) of effective symptoms control. Up to 73.3% (CSAI) and 80% (LCIG) of surveyed patients would recommend the therapy. Patients’ opinions in more detail are presented in Table 2.

### 3.2. The Subgroup of Patients Qualified for Both Infusion Therapies

The subgroup of patients who might choose between two infusion therapies included 31 patients, 12 (38.7%) treated with CSAI and 19 (61.3%) with LCIG. The CSAI group counted 8 (66.7%) males, while among LCIG patients, there were 10 (52.6%) male patients; however, there were no statistical differences between genders in both groups. The mean age of the patients was 69.4 ±4.6 (CSAI) and 71.1 ± 8 (LCIG) years, respectively (Table 3). The median disease duration was 16 years (12–19) for CSAI and 15 (14–19) years for LCIG. The median time of DATs was 1 (0–3) (CSAI) and 3 (2–3.5) (LCIG) years. The mean MDS-UPDRS part III for CSAI users at the moment of qualification to therapy was 52.3 ± 16 (OFF) and 28.3 ± 12.5 (ON). In the LCIG group, there were 52.0 ± 13.6 and 26.6 ± 9.1 for the OFF and ON states, respectively. The questionnaire was conducted with the family/caregiver and with a patient. For the vast majority, patients reported being in relationships (83.3% CSAI, 78.9% LCIG) and living with a family (83.3% CSAI, 89.5% LCIG). Most patients required assistance in operating the device used for the treatment. Only 16.7% CSAI and 31.6% LCIG users declared fully self-administering the pomp. Furthermore, patients reported using additional oral medication, respectively, 83.3% of CSAI patients and 79% in the LCIG group. Detailed characteristics of groups are presented in Table 3.

Patients who were qualified for both CSAI and LCIG presented different preferences about the therapy. The most relevant factor differentiating was fear of surgical procedure (9 (75%) vs. 7 (36.8%), *p* = 0.043) and the safety of therapy (10 (83.3%) vs. 9 (47.4%), *p* = 0.049), which gained statistical significance. Both groups displayed trust in the doctor (100% of CSAI, 94.7% of LCIG patients), and the information provided by the doctor (100% of CASI, 89.5% of LCIG patients) was crucial. The least important factor for the patients was the aesthetics of the device (0% of CSAI and 5.3% of LCIG patients). The summary of the factors influencing the choice and preferences of therapy is shown in Table 4.

## 4. Discussion

In our study, a total of 50 patients treated with infusion therapies were evaluated on their satisfaction and opinion about DAT therapy. Moreover, 31 people were assessed on their preferences between CSAI and LCIG.

The results of the research displayed some significant differences between infusion therapies which need to be considered during the selection of the proper method. LCIG patients reported longer control of parkinsonian symptoms than the CSAI patients. LCIG is an effective form of treatment in reducing OFF states [13]; however, our results showed that patients treated with LCIG presented higher PDQ-8 scores than patients with CSAI. Some literature data indicate that this treatment significantly enhances the quality of life [16,17]. Antonini et al. showed in their meta-analysis the significantly greater improvement in the quality of life for LCIG patients compared to CSAI after 6 months of therapy using both methods [17]. However, the mentioned studies had a different design and follow-up assessment. Our study had a cross-sectional character and was focused on the possibility of choice between therapies and patient preferences. Differences may be associated with the choice of less invasive methods by CSAI patients due to fear of surgery affecting their psychological comfort. Furthermore, 75% of CSAI patients reported this factor as relevant in the decision-making process. Aydemir et al. presented results indicating that 61.4% of PD patients were concerned about surgical procedures, and that was the most common reason for declining STN-DBS or LCIG by the patients [11]. Similar results were gained by Marshall et al., who showed that patients preferred pomp therapy over the STN-DBS therapy, but the possibility of tube replacement concerned the patients as well [18]. The invasiveness of LCIG poses a significant disadvantage of this method. For patients who value the safety of therapy, a better choice seems to be CSAI. However, the differences in quality of life assessment need to be addressed in further study to be explained.

Another observation seems to be the caregiver’s burden. In our study, the total score of BSFC-s for LCIG was almost twice as high as for CSAI. It may be associated with connecting and disconnecting the pump every day, cleaning the tube, and skin care as well [12]. It seems relevant to pay attention to family caregivers’ opinions in selecting the method.

Taking into consideration the advantages and disadvantages of both infusion methods, an innovative solution seems to be applying LDP/CDP [19], which combines the strengths of CSAI and LCIG. A subcutaneous application is easier and definitely less invasive, which consequently may decrease caregiver burden. The surgery is not required as well. As our results commanded, the safety of therapy is relevant for patients. Thus, the subcutaneous foslevodopa/foscarbidopa therapy may be the answer to patients’ needs. At the time of this study was conducted, LDP/CDP was not available on the Polish market, but it was introduced right after the study was completed (1 July 2024).

Considering the different specificity of infusion therapies compared to DBS, our research described only patients previously disqualified from DBS or those who preferred infusion therapies. In the study group, 33 (66%) patients had contraindications, where the main obstacle was major cognitive impairment. Aydamir et al. described 19 from a group of 58 people who could not use DBS therapy, similarly, most of them are due to dementia syndrome [11]. It seems to be a relevant disadvantage of DBS.

Due to advancements in diagnostics and technology, there is a growing population of PD patients who may benefit from DATs. This not only involves the need to establish easy ways to qualify patients for DATs from a clinician’s perspective [20], but also a great need for clinicians to discuss the advantages and disadvantages of possible DATs with patients and their caregivers during the qualification process. Personalizing the treatment approach is crucial [8,9,21], particularly in light of the expanding knowledge about PD, including its genetic underpinnings [22]. Future investigations should focus on a larger, more diverse population and explore newly introduced therapies like LDP/CDP, which may offer a balanced alternative by combining the strengths of both CSAI and LCIG. Additionally, the relevant matter demanding detailed research seems to be the impact exerted by infusion therapies on caregivers’ mental health and quality of life.

We are aware that our study had several limitations. First of all, a small study group influences statistical significance. On the other hand, the research presented was a survey study which is associated with subjective data. Nevertheless, the study assessed only infusion therapies, which was intentional. However, it may limit our results.

## 5. Conclusions

Given the increasing prevalence of PD, the results of this study provide clinically valuable insights into the decision-making process for infusion therapies. The most important factors that patients considered when choosing a therapy were their safety and invasiveness. Patients highly value the trust and information provided by doctors, who can help select the most appropriate treatment for each individual. These findings may inspire more personalized, patient-centered approaches to managing PD, potentially enhancing treatment adherence and outcomes. Furthermore, understanding the distinct advantages and disadvantages of CSAI and LCIG, as highlighted by patient preferences, may help clinicians tailor treatment plans that align with individual needs, particularly concerning medication reduction, symptom control, and caregiver burden to optimize the effectiveness and satisfaction of these treatment methods.

## Figures and Tables

**Table 1:** Characteristic of the whole study group.

	CSAI	LCIG	*p*-Value
Number of patients, *n* (%)	15 (30)	35 (70)	
Gender, males, *n* (%)	9 (60)	19 (54.3)	0.709
Mean age, years	70 ± 4.7	71 ± 2	0.742
Median disease duration, years	15 (12–19)	18 (13–19)	0.401
DATs time of use, years	1 (1–4)	2 (1–4)	0.382
Mean MDS-UPDRS part III OFF	52.9 ± 14.4	56.9 ± 15.0	0.381
Mean MDS-UPDRS part III ON	29.7 ± 12.2	29.7 ± 9.8	0.995
Married/in a relationship, *n* (%)	12 (80)	28 (80)	0.659
Live with, *n* (%)			0.378
Family	12 (80)	31 (88.6)	
Caregivers	1 (6.7)	0	
Alone	2 (13.3)	3 (8.6)	
In rest home	0	1 (2.9)	
Education, *n* (%)			0.793
Higher education	4 (26.7)	6 (17.1)	
Secondary education	6 (40)	13 (37.1)	
Professional education	4 (26.7)	14 (40)	
Basic education	1 (6.7)	2 (5.7)	
Operation device, *n* (%)			0.970
Only a caregiver	4 (26.7)	11 (31.4)	
Mainly a caregiver	3 (20)	6 (17.1)	
Patient with help	5 (33.3)	10 (28.6)	
Patient self-sufficiently	3 (20)	8 (22.9)	
Taking oral antiparkinsonian treatment in addition to DAT *, *n* (%)			0.348
Yes	12 (80)	31 (88.6)	
No	3 (20)	4 (11.4)	
PDQ-8 mean total score	**13 (6–19)**	**20 (13–27)**	**0.008**
BSFC-s mean total score	**9 (2.5–13)**	**19 (12–21)**	**0.011**

Statistical differences were analyzed using the Chi^2^ test with exact Fisher’s test for qualitative variables. For the results, *p* < 0.05 post hoc analysis was performed. For quantitative variables, the U-Mann–Whitney test or Student’s t-test after the normality of distribution was determined. The mean values are reported with standard deviations. The median values are reported with the interquartile range. The statistically significant results have been bolded. * Antiparkinsonian treatment included levodopa and dopamine agonists. Abbreviations: continuous subcutaneous apomorphine infusion (CSAI), levodopa–carbidopa intestinal gel (LCIG), Movement Disorder Society-Unified Parkinson’s Disease Rating Scale (MDS-UPDRS), device-aided therapy (DAT), Short Version of the Burden Scale for Family Caregivers (BSFC-s), Parkinson’s Disease Questionnaire-8 (PDQ-8).

**Table 2:** Opinion of the patients with Parkinson’s disease using infusion therapies (CSAI and LCIG).

	CSAI, *n* (%)	LCIG, *n* (%)	*p*-Value
Number of patients, *n* (%)	15 (30)	35 (70)	
Does the treatment provide sufficient control of the symptoms of the disease?			0.600
Yes	9 (60)	26 (74.3)	
No	4 (26.7)	6 (17.1)	
Do not know	2 (13.3)	3 (8.6)	
How many hours a day does the current treatment effectively reduce symptoms of the disease?			**0.023**
10–16 h	7 (46.7)	14 (40)	0.448
6–10 h	**1 (6.7)**	**13 (37.1)**	**0.026**
4–6 h	3 (20)	7 (20)	0.660
<4 h	**4 (26.7)**	**1 (2.9)**	**0.024**
Would you recommend your method of therapy?			0.404
Yes	11 (73.3)	28 (80)	
No	0	2 (5.7)	
Do not know	4 (26.7)	5 (14.3)	
Would you like to end your current therapy?			0.959
Yes	2 (13.3)	4 (11.4)	
No	11 (73.3)	27 (77.1)	
Do not know	2 (13.3)	4 (11.4)	

Statistical differences were analyzed using Chi^2^ test with the exact Fisher’s test. For the results, *p* < 0.05 post hoc analysis was performed. The statistically significant results have been bolded. Abbreviations: continuous subcutaneous apomorphine infusion (CSAI), levodopa–carbidopa intestinal gel (LCIG).

**Table 3:** Characteristics of the patients who might choose between two infusion therapies.

	CSAI	LCIG	*p*-Value
Number of patients, *n* (%)	12 (38.7)	19 (61.3)	
Gender, males	8 (66.7)	10 (52.6)	0.347
Mean age, years	69.4 ± 4.6	71.1 ± 8	0.552
Median disease duration, years	16 (12–19)	15 (14–19)	0.902
DATs time of use, years	1 (0–3)	3 (2–3.5)	0.220
Mean MDS-UPDRS part III OFF	52.3 ± 16	52.0 ± 13.6	0.951
Mean MDS-UPDRS part III ON	28.3 ± 12.5	26.6 ± 9.1	0.669
Married/in relationship, *n* (%)	10 (83.3)	15 (78.9)	0.574
Live with *n* (%)			0.438
Family	10 (83.3)	17 (89.5)	
Caregivers	1 (8.3)	0	
Alone	1 (8.3)	2 (10.5)	
Education, *n* (%)			0.987
Higher education	2 (16.7)	3 (15.8)	
Secondary education	5 (41.7)	8 (42.1)	
Professional education	4 (33.3)	7 (36.8)	
Basic education	1 (8.3)	1 (5.3)	
Operation device, *n* (%)			0.806
Only a caregiver	4 (33.3)	6 (31.6)	
Mainly a caregiver	2 (16.7)	2 (10.5)	
Patient with help	4 (33.3)	5 (26.3)	
Patient self-sufficiently	2 (16.7)	6 (31.6)	
Taking antiparkinsonian treatment in addition to DAT *			0.574
Yes	10 (83.3)	15 (79)	
No	2 (16.7)	4 (21)	

Statistical differences were analyzed using the Chi^2^ test with exact Fisher’s test for qualitative variables. For the results, *p* < 0.05 post hoc analysis was performed. For quantitative variables, the U-Mann–Whitney test or Student’s t-test after the normality of distribution was determined. The mean values are reported with standard deviations. The median values are reported with the interquartile range. * Antiparkinsonian treatment included levodopa and dopamine agonists. Abbreviations: device-aided therapy (DAT), continuous subcutaneous apomorphine infusion (CSAI), levodopa–carbidopa intestinal gel (LCIG), Movement Disorder Society-Unified Parkinson’s Disease Rating Scale (MDS-UPDRS).

**Table 4:** Factors influencing the choice and preferences of therapy of the patients with advanced Parkinson’s disease.

Factor	CSAI, *n* (%)	LCIG, *n* (%)	*p*-Value
Aesthetics of the device	0	1 (5.3)	0.613
Complicated operation of device	4 (33.3)	3 (15.8)	0.241
Limited help from family/caregivers	2 (16.7)	1 (5.3)	0.328
Desire to be self-sufficient	6 (50)	12 (63.2)	0.362
Fear of surgery	**9 (75)**	**7 (36.8)**	**0.043**
Effectiveness of therapy	8 (66.7)	15 (78.9)	0.362
Safety of therapy	**10 (83.3)**	**9 (47.4)**	**0.049**
Trust in the doctor	12 (100)	18 (94.7)	0.612
Information provided by the doctor	12 (100)	17 (89.5)	0.368
Information provided by other patients	3 (25)	4 (21.1)	0.565
Information from the Internet	5 (41.7)	7 (36.8)	0.541
Reputation of the Hospital	7 (58.3)	9 (47.4)	0.411

Statistical differences were analyzed using Chi^2^ test with the exact Fisher’s test. The statistically significant results have been bolded. Abbreviations: continuous subcutaneous apomorphine infusion (CSAI), levodopa–carbidopa intestinal gel (LCIG).

## Data Availability

Data are available by correspondence.

## Supplementary Materials

The following supporting information can be downloaded at: https://www.mdpi.com/article/10.3390/medicina61010027/s1.
