# Supplementary material for: Satisfaction and Preferences for Infusion Therapies in Advanced Parkinson’s Disease—Patient Perspective
Source: Medicina (Kaunas). 2024 Dec 28;61(1):27. doi: 10.3390/medicina61010027 (PMC11766721; doi:10.3390/medicina61010027)
Supplement: Supplementary file 1 [file medicina-61-00027-s001.zip › medicina-3364041-supplementary.pdf]

Satisfaction and preferences for infusion therapies in advanced Parkinson's disease – the survey

DEMOGRAPHIC AND INFUSION THERAPY DATA

1. Gender:
    a. Female
    b. Male
2. Age: …
3. Education:
    a. Primary
    b. Secondary
    c. Vocational
    d. Higher education
4. Marital status:
    a. Married/in relationship
    b. Single
5. Living with:
    a. Alone
    b. Family
    c. Caregiver
    d. Rest home
6. Which of infusion therapies have you use currently?
    a. levodopa-carbidopa intestinal gel (LCIG)
    b. continuous subcutaneous apomorphine infusion (CSAI)
7. Who operates the pump?
    a. I operate the pump myself
    b. I operate the pump with the help of family/caregiver
    c. The family/caregiver mainly operates the pump
    d. Only the family/caregiver operates the pump
8. Do you take oral antiparkinsonian medications?

a. Yes

b. No

ASSESSMENT OF PATIENT'S PREFERENCES

9. What factors were important to you when choosing a treatment method (choose all important)?*

Aesthetics of the device	
Complicated operation of the device	
Limited help from family/caregivers	
Desire to be self-sufficient	
Fear of surgery	
Effectiveness of therapy	
Safety of therapy	
Trust in the treating physician	
Information provided by the doctor	
Information provided by other patients	
Information from the Internet	
Reputation of the Hospital	

10. Does the current method provide satisfactory symptom control?

    a. Yes, I would

    b. No, I would not

    c. I do not know

11. How many hours per day when treatment effectively suppress symptoms?

    a. 10-16 hours per day

    b. 6-10 hours per day
    c. 4-6 hours per day
    d. Up to 4 hours per day
12. Would you recommend the treatment method used?
    a. Yes, I would
    b. No, I would not
    c. I do not know
13. Would you like to end your current therapy?
    a. Yes, I would
    b. No, I would not
    c. I do not know

*The question is only for patients who could choose the therapy.
